# Cyberbiosecurity in Advanced Manufacturing Models

**DOI:** 10.3389/fbioe.2019.00210

**Published:** 2019-09-04

**Authors:** Donovan Guttieres, Shannon Stewart, Jacqueline Wolfrum, Stacy L. Springs

**Affiliations:** Center for Biomedical Innovation, Massachusetts Institute of Technology, Cambridge, MA, United States

**Keywords:** cybersecurity, biomanufacturing, distributed manufacturing, bioprocess risks, cyber-physical systems, risk mitigation

## Abstract

Cybersecurity for the production of safe and effective biopharmaceuticals requires the attention of multiple stakeholders, including industry, governments, and healthcare providers. Cyberbiosecurity breaches could directly impact patients, from compromised data privacy to disruptions in production that jeopardize global pandemic response. Maintaining cybersecurity in the modern economy, where advanced manufacturing technologies and digital strategies are becoming the norm, is a significant challenge. Here, we highlight vulnerabilities in present and future biomanufacturing paradigms given the dependence of this industry sector on proprietary intellectual property, cyber-physical systems, and government-regulated production environments, as well as movement toward advanced manufacturing models. Specifically, we (1) present an analysis of digital information flow in a typical biopharmaceutical manufacturing value chain; (2) consider the potential cyberbiosecurity risks that might emerge from advanced manufacturing models such as continuous and distributed systems; and (3) provide recommendations for risk mitigation. While advanced manufacturing models hold the potential for reducing costs and increasing access to more personalized therapies, the evolving landscape of the biopharmaceutical enterprise has led to growing concerns over potential cyber attacks. Gaining better foresight on potential risks is key for implementing proactive defensive principles, framing new developments, and establishing a permanent security culture that adapts to new challenges while maintaining the transparency required for regulated production of safe and effective medicines.

## Introduction

Cybersecurity attacks and data breaches are a matter of when, not if, with companies in all sectors and of all sizes vulnerable. Between 2014 and 2015, the Federal Bureau of Investigation (FBI) reported a 53% increase in incidents of industrial or economic espionage targeted at the U.S (Barrett, 2015). In the healthcare industry, data collected by the Department of Health and Human Services shows a 10% increase in the number of reported incidents each year since 2010 (U S Department of Health and Human Services Office for CivilRights, 2019). In 2017, this industry accounted for 18% of all data breaches, with 63% of incidents caused by criminal or malicious activity.

More recently, cyber extortionists have targeted hospital IT systems, successfully extracting thousands of dollars in ransoms because of the critical and often time-sensitive nature of the information (Osborne, 2018). In one example, the WannaCry ransomware affected hospitals within UK's National Health Service, leading to 6,912 medical appointment cancellations and 1,220 pieces of IT-connected diagnostic equipment infected, largely due to unpatched or unsupported Windows operating systems (National Audit Office, 2018). Increasing concerns over the potential threat cyberattacks can have on acquiring access to and control of medical devices, especially if digitally connected (e.g., insulin pumps) or on a hospital network (e.g., radiologic imaging equipment), has led the FDA to release pre- and post-market guidance to reduce cyber-related risks (U. S. Federal Drug Administration, 2018). Spaces such as the Biohacking Village (https://www.villageb.io/) encourage dialogue between medical device and cybersecurity professionals, but should also consider biomanufacturing-related vulnerabilities.

## New Risks Within the Growing Bioeconomy

The bioeconomy has become a principal driver of national GDP (National Academies of Sciences Engineering and Medicine, 2015). Nevertheless, there is insufficient attention and collection of data on the risk of cyberattacks targeting organizations that manufacture life-saving or -extending biologic medicines, such as vaccines, recombinant proteins, monoclonal antibodies, and advanced therapy medicinal products (ATMPs). Whether the biotechnology industry is deliberately targeted or collateral damage in cyber warfare, the effects could be severe given the high-value products and data involved.

The intellectual property, manufacturing processes, regulatory requirements and sophisticated cyber-physical systems involved in the production of biologic therapies may be particularly vulnerable to three major forms of cyberattacks: sabotage (deliberate and malicious acts that damage digital or physical infrastructure), corporate espionage (gaining access to sensitive information to attain advantage over an adversary), and crime/extortion (encrypting files with a ransom note asking for remuneration for their return) (Morag, 2014). Examples of each have been reported across the biotechnology industry (Panda Security., 2017; Sackner-Bernstein, 2017; Symantec., 2018). Despite differences in these modes of cyberattack, their mechanisms can often be similar (e.g., phishing attacks, malware, encryption blind spots, cloud-based threats, negligence, and poor institutional knowledge of risks). In all their forms, cybersecurity incidents raise serious concern for the biopharmaceutical industry, government, regulators, health service providers and ultimately patients.

The formalization of cyber*bio*security, at the nexus of cybersecurity, cyber-physical security and biosecurity as applied to biological and biomedical-based systems, provides insight into the unique risks present in the biotechnology industry (Murch et al., 2018). More specifically, biopharmaceutical companies employ cyber-physical systems across a range of functions: raw materials sourcing, cell line development and optimization, upstream and downstream process development, manufacturing, validation studies, clinical trials, supply chain management of products, post-market drug safety monitoring, and interfacing with health providers. Process control strategies increasingly collect and use data to ensure that manufacturing processes meet product quality standards. As part of advanced manufacturing approaches, various tools (e.g., internet-of-things, artificial intelligence) are allowing for more responsive control to optimize for reproducibility, quality, safety and supply (Helu and Hedberg, 2015; Zhong et al., 2017). However, in-line, at-line or remote data monitoring can also increase vulnerability to cyberattacks given the increasing reliance on digital and automated control systems (Babiceanu and Seker, 2016).

## Known Cybersecurity Risks Point to Vulnerabilities in Biomanufacturing

U.S. biopharmaceutical companies together spend nearly $160 billion each year on R&D, and their accumulated intellectual property (IP) is likely worth trillions of USD (Research America., 2016). An advanced, persistent attack could allow corporate rivals to steal internal communications, IP related to the product or process, and facility monitoring data to gain a competitive advantage. A malware program called Dragonfly specifically targets cyber-physical systems used in pharmaceutical manufacturing equipment, stealing trade and manufacturing secrets as a form of corporate espionage (Carman, 2014). Some have suggested that Dragonfly could also be used for physical sabotage in the future (Symantec Security Response., 2014). Pharmaceutical companies hold patient data related to clinical trials and disease management in their corporate networks. Since the data is both highly sensitive personal information and regulated, breaches can both incur large fines and damage a firm's reputation. Assessing emerging cybersecurity risks across the biopharmaceutical industry is especially important and timely as many companies work to establish digital strategies and data lakes that serve as repositories of data from across company functions (e.g., drug discovery and development, process design, manufacturing, quality control, clinical trials, real-world evidence). While such systems can help centralize large amounts of information, there are increasing concerns over data security and concentrating risks on a single network.

**Case Example - Merck & Co.:** In June of 2017, the biopharmaceutical company Merck & Co. was affected by the malicious worm NotPetya (Erman and Finkle, 2017). The worm was based on ransomware, Petya, but it had been modified so that it was unable to revert its changes, resulting in the permanent encryption of data (Goodin, 2017). Since the malware affected computer systems that are used to control Merck's manufacturing process, the attack resulted in shortages of the Gardasil vaccine and may have contributed to stock-outs of the Hepatitis B vaccine. The incident led Merck to borrow $240 million worth of Gardasil vaccine from the Center for Disease Control's stockpile, with a total estimated cost of the cyberattack close to $1 billion (United States Securities and Exchange Commission, 2018). In February 2018, the US and UK publicly attributed the attack to Russia (Marsh, 2018). Since there is no evidence to believe that Merck had been deliberately targeted, it is easy to imagine a more tailored or intentional cyberattack causing even more damage to biomanufacturing activities. Given the low number of reported cases of cyberattacks impacting biomanufacturing processes, learning from this experience is of paramount importance. More recently, Roche and Bayer reported cyberattacks from the Winnti malware attributed to hackers in China, but were both able to detect the attack before any sensitive information could be stolen (Rees, 2019).

The risks and implications of cyberbiosecurity events, such as in the case of Merck & Co., may be underappreciated, especially as the role of biologic therapies across a range of conditions becomes increasingly important for meeting healthcare needs. From a manufacturing perspective, the consequences include occupational hazards, damage to equipment, batch failure leading to loss of product, and theft of IP. Regulatory burden could increase as manufacturers are required to re-establish compliance after cyberattacks, re-qualify equipment or re-validate processes. Shortages or stock-outs of medicines can lead to a loss of public trust in institutions like hospitals or the pharmaceutical industry, as well as financial burden (Caulder et al., 2015). From a patient perspective, interruptions in the supply of biologic medicines could be life threatening. The potential consequences of a cybersecurity breach range from sudden, catastrophic events such as a plant shutdown to subtler deviations in quality that introduces hard-to-detect risks into the process and increases likelihood of lot failure.

The biopharmaceutical industry is generally considered a high-value, capital-intensive and critical industry, making it an attractive target for extortionists. The batch production model for biologic therapies, vaccines and recombinant proteins, in particular, physically concentrates revenue centers since production takes place at large scales. This makes the industry vulnerable, as companies may have few runs throughout the year that each last several weeks and any form of interruption in production can damage a significant fraction of the yearly output.

While large stainless steel bioreactors have been the industry standard, there is a shift toward more flexible, single use systems that enable faster turnaround and response to uncertain demand, especially as precision medicines become available for smaller patient populations. As the industry considers more advanced manufacturing models (e.g., continuous manufacturing, real-time feedback control, etc.), close attention must be paid to the principles of information security. To identify interventions that can build resilience against potential cybersecurity threats, vulnerabilities in today's manufacturing operations, as well as future operational settings, need to be more closely examined.

## Digital Information Flow in Biomanufacturing

Information exchange of highly sensitive data can be seen across the entire biomanufacturing value chain. Since a typical biomanufacturing company and the corporate network (e.g., vendors, contract manufacturing organizations) it operates in have numerous possible vulnerabilities, an important first step for these organizations is mapping risks. The following is a general, though not exhaustive, schema to help identify possible cyberattack vulnerabilities of a biomanufacturing facility. Organizations should engage experts to determine their individual security needs as they pertain to unique product types, manufacturing requirements, patient populations, regulatory jurisdictions, and geographies.

**Figure 1:**
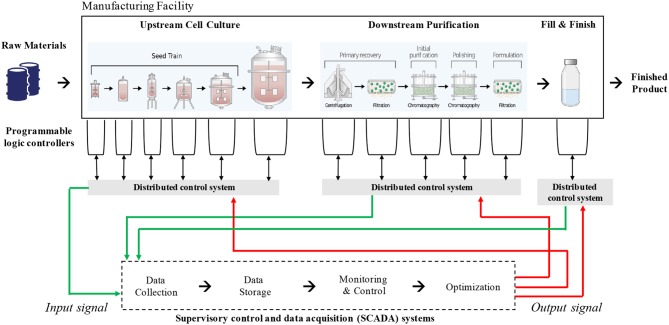
Information flow in typical biomanufacturing operations for the production of monoclonal antibodies. The schematic indicates various cyber-physical systems that interact with each other to maintain process control. The data generated and processed by the SCADA is largely confined within a manufacturing facility.

A typical biomanufacturing plant makes use of a wide range of cyber-physical systems such as sensors, actuators, programmable logic controllers (PLCs), distributed control systems (DCSs), and (in some cases) supervisory control and data acquisition (SCADA) systems (Sokolov et al., 2017). Sensors and actuators are the electronic components that take measurements of specific parameters (e.g., pH, liquid level) and execute physical responses, such as opening valves or starting or stopping a physical process. These systems are often dictated by mathematical models and algorithms with pre-determined responses based on measurements. Figure 1 illustrates the role these systems play within standard biomanufacturing operations for the production of monoclonal antibodies.

PLCs are interfaces between specialized machinery, such as bioreactors or chromatography skids, and users. They often have specialized operating systems, sometimes with limited input interfaces, that allow them to perform dedicated functions such as integrating and displaying sensor information. They may also give feedback or automated commands that cause the system to continuously perform within preprogrammed parameters such as temperature, gas saturation, or solvent mix. PLCs have previously been overlooked in cybersecurity plans, with little awareness from manufacturers that controllers directly connected to the internet are all searchable using a single search engine, SHODAN (Wang et al., 2015). Alarmingly, SHODAN allows searchers to easily filter by machines that have retained their default security credentials. In 2011, the US Department of Homeland Security issued warnings through the Industrial Control Systems Cyber Emergency Response Team (ICS-CERT) that nearly all PLCs are vulnerable to hackers. For instance, attackers may cause sensors to report false data or modify algorithms in control systems in ways that can jeopardize product quality, damage manufacturing equipment, and potentially induce occupational hazards.

DCSs are becoming increasingly important to the overall functioning of the production line, where multiple systems have to be coordinated to achieve the desired finished product while preventing waste or accident. They may display and integrate data from PLCs, hold plans or models, perform calculations, and allow supervisory control via input from plant workers. The DCSs allows the system or human supervisors to execute controls that affect the speed and quality of production. Because these systems can include multipurpose computers, they contain a rich amount of organizational data, and they are vulnerable to the wide array of cyberattack vectors that can affect any cyber-physical system. Some specialized hardware components for these systems could take months to replace if damaged, while re-qualifying equipment or re-validating processes can lead to lengthy supply disruptions.

A SCADA system is used in complex or distributed manufacturing systems, where a central command center issues controls and receives feedback from remote sites where physical manufacturing processes are taking place. This type of network is used in biomanufacturing for large, complex operations, or in cases where manufacturing needs to take place close to the point of care. These networks not only integrate information from the plant itself, but may also tie in to supply chain logistics or transmit information over large distances on the internet.

## Next Generation Manufacturing

The biomanufacturing landscape is rapidly changing, partly due to technological advancements leading to process intensification, miniaturization, and automation, as well as to increased digitization of process controls more generally.

### Continuous Manufacturing

Continuous manufacturing allows for a fully automated end-to-end assembly line from raw materials to products, compared to traditional batch manufacturing that requires intervention between steps of the process. Under this manufacturing paradigm, raw materials are fed into a process train and finished products removed from the other end in a continuous manner. These allow for more control over process parameters and can be run 24/7 to reduce production time. Continuous manufacturing may present additional benefits such as reducing the likelihood of costly batch dumping and real-time release of final product (National Academies of Sciences Engineering Medicine, 2019). Nevertheless, these systems present new challenges with regards to regulatory compliance and increased reliance on sensor technology for analysis of critical quality attributes. The uptake of continuous manufacturing is leading to smaller-footprint facilities with lower capital costs and thus further promote decentralization of production.

### Distributed Manufacturing

Historically, the biopharmaceutical industry has concentrated manufacturing of biologics to one or few geographic locations to take advantage of economies of scale and make up for the large capital investment required in stainless-steel plants. More recently, there has been growing interest toward dividing production across multiple sites or geographic regions. A shift toward distributed manufacturing has partially been driven by the need for production systems that are more responsive to changing demand and patient-specific needs. More distributed systems, enabled by single-use technology and other advances in biomanufacturing, will make facilities more flexible and modular. Such systems rely increasingly on automation and digital networks to ensure replicability of manufacturing quality across sites, while reducing delivery time of products. While offering these potential benefits, they also introduce complex organizational and regulatory challenges, especially with regards to the cybersecurity of increasingly connected digital systems (Harrison et al., 2018).

## Considerations for Emerging Biologic Products

ATMPs are unique in that they can require a high level of personalization and customization that could make large-scale manufacturing impractical. For these therapies, cells can either be harvested from a patient, modified, and returned to the patient (autologous) or cells originating from a single donor provide treatments to large numbers of patients (allogeneic). While the recent approvals of chimeric antigen receptor-modified T cells (CAR T) spur investment into the development and production of ATMPs for a variety of new indications, manufacturing processes are not currently optimal and will likely evolve in coming years. Unique manufacturing challenges arise due to patient-specific requirements, input material variability, process-related features, and short shelf-life, amongst other factors. The production of autologous cell therapies, for example, in both a centralized and distributed manufacturing model makes use of a more complex digital information flow than that presented in Figure 1.

**Figure 2:**
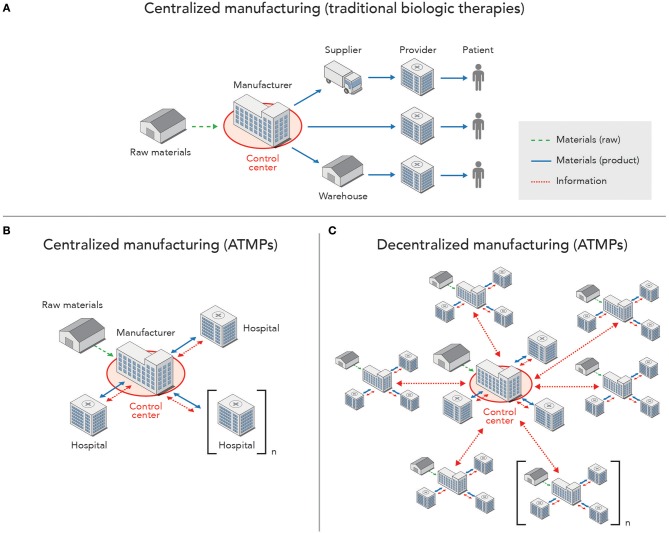
High-level representation of information flow, including raw materials and finished products, across a network of manufacturing sites, patient providers, and control centers for monitoring of SCADA systems. **(A)** Centralized manufacturing of traditional biologic products, such as monoclonal antibodies as indicated in Figure 1, with a unidirectional flow of materials from the manufacturing facility to providers (e.g., pharmacies, hospitals, retailers), while information on the product is tracked across the supply chain, and a control center is housed within the facility. **(B)** Centralized manufacturing of ATMPs, which requires information and material exchange with each patient found in one or more hospitals, while the control center is still housed within the manufacturing facility. **(C)** Decentralized production of ATMPs with multiple manufacturing sites that each interface with a control center (located within one of the manufacturing facilities in the network or standalone) that monitors and manages the SCADA systems found in each facility.

The network of facilities involved in the production and distribution of biologic therapies, as well as flow of information (data, raw materials and finished products) between them is shown in Figure 2, which maps both traditional biopharmaceutical and advanced (e.g., ATMP) manufacturing. Production of increasingly personalized therapies using advanced manufacturing models leads to more complex exchange of information and materials that may make these activities more susceptible to interruptions. Clinics serve as the starting point for collecting cells through apheresis and endpoint for infusion of the final product. In between, patient-specific input materials are transported to a centralized or separate manufacturing units, each with a complex set of cyber-physical systems that maintain process control. Information exchange across the network (e.g., patient, clinic, manufacturing site(s), supply chain) demonstrates the added physical and digital complexity for manufacturing these emerging therapies, thus increasing vulnerability to cyberattacks.

Cyber-physical systems that automate manufacturing steps will play an important role in ensuring well-controlled, consistent processes while maintaining high drug quality through centralized control centers that aggregate and analyze data to inform decisions. These therapies involve complex logistics for the collection and delivery of cells to and from patients, with tight turnaround times and coordination of activities at the clinical and manufacturing sites. The need for batch release close to real-time will likely lead to additional automated procedures for validation of product quality. Data continues to be important for assurance of product quality, but there is need to better integrate pre-process, in-process and release data to execute controls across all sites and not just the one where an observation arises (Harrison et al., 2017).

Increasing use of digital systems for ATMPs, whether to monitor product quality or manage data across the product value chain, brings with it the risk of further exposing manufacturing systems to cyberattacks. These manufacturing networks feature geographically-distributed signal input and output, multiple distributed human-machine interfaces, and often, explicit tracking and use of patient data. The increasing amount and type of transmitted data opens up opportunities for malicious attackers to steal sensitive information such as patient or process information and extort money through ransomware. Additionally, high levels of variability expected in data coming from numerous distributed production systems may make it difficult to detect more subtle risks and intrusions from cyberattacks.

## Ensuring a Resilient Biomanufacturing Industry

The biopharmaceutical manufacturing industry continues to be vulnerable to cyberattacks because of the prevailing misconception that cybersecurity concerns can be dealt with using IT solutions alone and from incomplete awareness of the type and level of perceived risks, as well as limited time and resources (Kalyvas et al., 2017). Additionally, small startups in the industry may be especially susceptible since they typically run with the leanest possible staffing and resources to address cybersecurity might be limited. However, as the Merck & Co. incident shows, highly connected industries can become collateral damage as worms travel indiscriminately across systems, so each company is only as secure as their most vulnerable partner.

What are some steps that the biopharmaceutical industry should consider? In response to the increased threat and economic impact of cyberattacks, in 2014 the National Institute of Standards and Technology (NIST) released a framework for improving the national cybersecurity infrastructure. Meant to address a broad range of cybersecurity risks and applicable to organizations of all sizes and all kinds, the framework structures its recommendations into a five-step plan: identify, protect, detect, respond, and recover (National Institute of Standards and Technology, 2014). Applying this framework to the unique risks faced by biomanufacturers, firms should first identify and map their potential attack surface, from corporate workstations to PLCs which can impact normal bioprocess operations. They should institute protections on all of these surfaces, such as implementing firewalls, changing default security credentials, encrypting sensitive information, and implementing available security features. To detect incidents in a timely fashion, organizations should implement intrusion detection systems and monitoring protocols. Organizations should also have emergency response plans in place with clear lines of command and reporting. Finally, they should give thought to their recovery strategy, including mapping where offline backups of critical data and system states are stored.

As advanced manufacturing systems are increasingly considered, both in response to cost pressures and due to the unique requirements of emerging therapeutic modalities, adopting and scaling a comprehensive cybersecurity plan will be a challenge. Attack surfaces are larger and exist in different forms across the information value chain, from process data interfaces to clinical data systems. With more units digitally connected, entry points can make the entire system vulnerable to attack. The tradeoffs that emerge when considering advanced manufacturing options (e.g., greater exposure to cyber threats vs. operational gains) indicate that next-generation manufacturing may be appropriate for some but not all applications and influenced by factors beyond manufacturing (e.g., corporate culture, geography). Therefore, special attention is needed to explore the unique changes ongoing in the biomanufacturing industry and the implications they will have on ensuring manufacturing security.

## Conclusion

Understanding the full spectrum of cybersecurity risks, including their relative likelihood and impact, across cyber-physical systems employed in biomanufacturing continues to be a challenge. This knowledge is important to proactively implement measures that will mitigate the risk and impact of cyberattacks. This requires a systematic approach to securitization, forward-looking and adaptive planning to best prepare for current and future risks, as well as promoting an industry-wide culture to address risks before they become emergencies. Suggestions have been made for greater investments in training employees, shifting the culture from one of loose self-regulation to heightened attention, and for industry to work more closely with regulators to design and implement safeguarding policies (Peccoud et al., 2018). With increasing use of complex models for advanced manufacturing, academia can play an important role in developing design principles and tools that can safeguard against cyberattacks.

Across the biomanufacturing industry, cyberattacks are experienced differently and few have been reported. Nevertheless, there are shared experiences and lessons learned that can make the entire industry safer and more resilient to a plethora of cybersecurity threats. Encouraging pre-competitive, multi-stakeholder collaboration on the best ways to prevent and detect multidimensional risks can promote knowledge sharing and improved security systems across the entire industry in ways that safeguard business interests and patient well-being. Since 2011, the Consortium on Adventitious Agent Contamination in Biomanufacturing (CAACB), a biopharmaceutical industry consortium housed at the Massachusetts Institute of Technology's Center for Biomedical Innovation, has worked to confidentially collect and anonymize data on virus contaminations in cell culture operations from Consortium-member companies. A similar approach could be taken to better understand and learn from cyberbiosecurity events across industry to move toward advanced manufacturing models in a united and safe way.

As the industry increasingly considers advanced manufacturing, especially for new therapeutic modalities, cyberbiosecurity needs to take a central role in in the design of digital strategies, business models, technologies, standards and regulations that ensure supply security. Emerging trends toward more continuous, single-use, and decentralized manufacturing will have unique implications, including unintended consequences, that will reshape the cyberbiosecurity landscape. Working together to build foresight on future potential risks will be key to turning uncertainties into opportunities in ways that safeguard biomanufacturing operations and improve access to care.

## Author Contributions

DG, SS, JW, and SLS all contributed equally to the design, research, writing, and review involved in the development of this manuscript.

### Conflict of Interest Statement

The authors declare that the research was conducted in the absence of any commercial or financial relationships that could be construed as a potential conflict of interest. The reviewer KL declared a past co-authorship with one of the authors SLS to the handling Editor.
